# Enhancing genetic disease control by selecting for lower host infectivity and susceptibility

**DOI:** 10.1038/s41437-018-0176-9

**Published:** 2019-01-16

**Authors:** Smaragda Tsairidou, O. Anacleto, J. A. Woolliams, A. Doeschl-Wilson

**Affiliations:** 1 The Roslin Institute and R(D)SVS, University of Edinburgh, Edinburgh, EH25 9RG UK; 2 Institute of Mathematical and Computer Sciences, University of São Paulo, São Paulo, Brazil

**Keywords:** Animal breeding, Agriculture

## Abstract

Infectious diseases have a huge impact on animal health, production and welfare, and human health. Understanding the role of host genetics in disease spread is important for developing disease control strategies that efficiently reduce infection incidence and risk of epidemics. While heritable variation in disease susceptibility has been targeted in livestock breeding, emerging evidence suggests that there is additional genetic variation in host infectivity, but the potential benefits of including infectivity into selection schemes are currently unknown. A Susceptible-Infected-Recovered epidemiological model incorporating polygenic genetic variation in both susceptibility and infectivity was combined with quantitative genetics selection theory to assess the non-linear impact of genetic selection on field measures of epidemic risk and severity. Response to 20 generations of selection was calculated in large simulated populations, exploring schemes differing in accuracy and intensity. Assuming moderate genetic variation in both traits, 50% selection on susceptibility required seven generations to reduce the basic reproductive number *R*_0_ from 7.64 to the critical threshold of <1, below which epidemics die out. Adding infectivity in the selection objective accelerated the decline towards *R*_0_ < 1, to 3 generations. Our results show that although genetic selection on susceptibility reduces disease risk and prevalence, the additional gain from selection on infectivity accelerates disease eradication and reduces more efficiently the risk of new outbreaks, while it alleviates delays generated by unfavourable correlations. In conclusion, host infectivity was found to be an important trait to target in future genetic studies and breeding schemes, to help reducing the occurrence and impact of epidemics.

## Introduction

Host genetic diversity affects infectious disease risk and impact (Keeling 1999; Doeschl-Wilson et al. 2011). Heritable genetic variation in susceptibility, i.e. an individual’s propensity of becoming infected when exposed to infectious material, is ubiquitous, and many genetic selection schemes in livestock and plants target reduction in host susceptibility (Heringstad et al. 2000; Kover and Schaal 2002; Bishop et al. 2010; Houston et al. 2010; Dos Santos et al. 2016; Banos et al. 2017). A second host trait affecting disease spread and severity that has been considered in epidemiology is the hosts’ infectivity (Read and Taylor 2001; Geenen et al. 2004; Keeling and Danon 2009; Brooks-Pollock et al. 2015), which refers to the ability of an individual, once infected, to transmit infection. In livestock breeding, individual variation in infectivity has not yet been exploited for disease control.

Individual variation in host infectivity has been observed in cases of super-spreaders, where a small fraction of highly infectious individuals generates disproportionally many new infections. Those have been described through the Pareto principle, where 20% of infected individuals are responsible for 80% of transmission events (Woolhouse et al. 1997; Lloyd-Smith et al. 2005). Super-spreading has been observed on a phenotypic level and is often attributed to social behaviour or heterogeneous contact structure. For example, in measles (Paunio et al. 1998), SARS and Ebola (Shen et al. 2004; Wong et al. 2015) in humans, in *Salmonella typhimurium* infection in mice (Gopinath et al. 2014), and in RNA-virus infections in bird species (Jankowski et al. 2013). The existence of super-spreaders has also been inferred in epidemiological models for bovine tuberculosis in cattle (O’hare et al. 2014). Super-spreading has often been attributed to characteristics of the pathogens rather than the hosts, for example in *Escherichia coli* infection in cattle (Chase-Topping et al. 2008). However, emerging evidence suggests that infectivity and super-spreading may be also partly controlled by host genetics (Geenen et al. 2004; Doeschl-Wilson et al. 2011, 2018; Lyall et al. 2011; Raszek et al. 2016; Anacleto et al. 2018). Those findings imply that individuals can evolve different disease response types affecting disease spread, and offer new opportunities for genetic disease control that go beyond the reduction of disease susceptibility (Doeschl-Wilson et al. 2018; Tsairidou et al. 2018b). By using individual genetic risk estimates for infectivity it may be possible to prevent or mitigate epidemics in livestock populations through reducing the presence of super-spreaders.

Epidemiological simulation models have shown that removal of super-spreaders would be an effective means for reducing epidemic severity (Lloyd-Smith et al. 2005). However, identification of highly infectious individuals prior to, or during the early stages of epidemics is extremely difficult in practice. Hence such models can realistically only predict the impact of removing super-spreaders as reactive disease control. In contrast, genetic disease control schemes are pro-active, i.e. preventive, as individuals can be selected based on genotypic information that can be collected at any stage, i.e. without the need of being exposed to infectious material. However, such genetic selection programmes usually operate on longer time-scales (over generations of selection) compared to those usually considered in epidemiological models. In epidemiology, the impact of realistic, long-term, pro-active genetic disease control, either alone or in combination with other control strategies, has rarely been assessed. The epidemiological benefits of genetic selection for reduced infectivity are thus currently unknown.

Until recently, estimating host genetic effects for infectivity from epidemiological data was not possible, as standard genetic evaluation tools routinely used in livestock industry do not fully capture genetic variance in infectivity (Lipschutz-Powell et al. 2012a, 2012b; Anche et al. 2015). Both susceptibility and infectivity are expressed through interactions between infected and non-infected individuals and, when subject to heritable variation, represent indirect genetic effects (IGE) (Lipschutz-Powell et al. 2012a; Anche et al. 2015; Baud et al. 2017). For IGEs, the phenotype of an individual depends on its own genetics and on the genetics of other individuals in the same contact-group. In other words, for infectious diseases, an individual’s infection status depends on its own genetic susceptibility and the genetic infectivity of its infected group-mates (which also depends on their genetic susceptibility). Hence, infectivity is not directly observable and on a phenotypic level is confounded with susceptibility, as both affect the infection status of group-members. Thus, infectivity and susceptibility are latent traits that need to be inferred through available, often incomplete, epidemic data. Conventional IGE models that have proved adequate for production traits (Bergsma et al. 2008; Bouwman et al. 2010), fail to capture the dynamic, non-linear nature of disease processes (Lipschutz-Powell et al. 2013; Anche et al. 2015; Biemans et al. 2017). However, recent breakthroughs in statistical inference methods can now provide reliable estimates of genetic effects for both infectivity and susceptibility from inferred infection times, without requiring direct observation of an ‘infectivity phenotype’ (Pooley 2014; Anacleto et al. 2015; Anche et al. 2015; Biemans et al. 2017). This implies that one can select directly on the traits that drive the epidemiology rather than on the observed infection status. With the necessary methodology developed, it is timely and relevant to consider the potential benefits arising from adding infectivity as a new disease phenotype into genetic analyses. In the context of livestock production, the question arises whether incorporating this additional phenotype, would make a sufficiently valuable contribution to current breeding schemes. In other words, what is the expected impact of additionally selecting for lower infectivity on future disease prevalence and possibility of disease eradication?

Genetic variation in IGEs has been shown to affect the magnitude and/or direction of response to selection in breeding schemes (Bijma et al. 2007; Ellen et al. 2007; Bergsma et al. 2008; Bijma and Wade 2008; Ødegård and Olesen 2011), and it has been suggested that exploiting IGEs in animal breeding can substantially increase the rate of genetic gain in the trait of interest (Ødegård et al. 2011; Anche et al. 2014b; Sae-Lim and Bijma 2016). However, the benefits of genetic selection for both reduced susceptibility and infectivity on practical epidemiological field measures, such as disease prevalence and duration of epidemics, that are commonly used for assessing economic losses and guide the development of effective (often multi-faceted) disease control strategies, are currently not known. This is because the relationship between host genetic susceptibility, infectivity, and the practical field outcomes of epidemics is non-linear. This non-linearity arises from the equations underlying the dynamic progression of epidemics over time, so that for example, a small change in susceptibility and infectivity can result in changing the expectation whether an epidemic will occur or not (Doeschl-Wilson et al. 2011). Therefore, the practical impact of combining both susceptibility and infectivity in selection schemes in one or more generations cannot be estimated by standard index theory, which assumes linearity, and additional epidemiological approaches are needed to model the dynamic, non-linear epidemic processes.

A key epidemiological measure for assessing the impact of disease interventions on epidemic risk and infection incidence is the basic reproductive ratio (*R*_0_); i.e. the expected number of secondary cases produced by a typical infected individual in a completely susceptible population (Diekmann et al. 1990). *R*_0_ has a threshold value of one which determines whether a disease outbreak can occur: when *R*_0_ is smaller than one, the epidemic will die out, whereas when *R*_0_ is greater than one major outbreaks can arise (Diekmann et al. 1990). Lipschutz-Powell et al. (2012a) suggested that selection on breeding values for direct and indirect effects reduces *R*_0_, while Anche et al. (2014a) theoretically demonstrated for single gene models that heritable variation in both host susceptibility and infectivity contribute to and can be utilised for reducing *R*_0._

The aim of this proof-of-concept study was to develop a quantitative modelling framework that predicts the benefits of genetic selection for reduced infectivity, in addition to reduced susceptibility, on epidemic risk and severity, and how these depend on the genetic variance, selection accuracy and intensity. For this purpose, a genetic–epidemiological model was developed to combine classical quantitative genetics theory with epidemiological prediction. Specifically, polygenic genetic variation in susceptibility and infectivity was incorporated in a stochastic epidemiological susceptible-infected-recovered (SIR) model to simulate epidemics in large livestock populations undergoing artificial genetic selection for lower susceptibility and infectivity, over several generations. The resulting model was then used to assess the efficacy of diverse genetic selection schemes targeting host susceptibility and infectivity in preventing disease outbreaks and reducing disease prevalence.

## Materials and methods

**Fig. 1:**
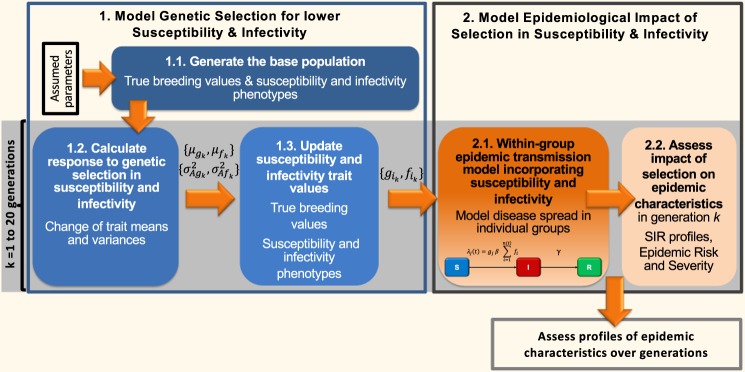
Modelling flowchart showing the different steps of the simulations. This process was replicated 50 times. Orange arrows indicate information flow

The effects of selection for lower susceptibility and infectivity on the risk and severity of epidemics were investigated on large simulated, genetically heterogeneous populations. The simulation process comprised two main parts (illustrated in Fig. 1): 1. modelling genetic selection for reduced susceptibility and infectivity, where populations were simulated for 20 generations based on selection with assumed accuracies and intensities; 2. modelling the epidemiological impact of selection by simulating epidemics in the populations of each generation generated in part 1, and assessing their epidemiological characteristics. For each scenario, 50 replicate simulations were conducted. Individual steps of each part are described in detail below:

### Modelling genetic selection for lower susceptibility and infectivity

Simulated populations of *N* = 10,000 half-sib individuals were generated in each generation. These were the progeny of 200 sires, each randomly mated to 50 dams, with all sires and dams unrelated, and each sire–dam combination producing one offspring. This structure is representative of livestock populations, e.g. dairy cattle population, where artificial insemination is practiced. Herein, susceptibility is defined as the propensity of becoming infected upon contact with an individual with average infectivity. Infectivity is defined as an individual’s ability, once infected, to transmit infection upon contact with a susceptible individual of average susceptibility (Anacleto et al. 2015). Susceptibility and infectivity are thus latent traits.

Latent susceptibility (*g*) and infectivity (*f*) were assumed to follow a log-normal distribution to accommodate the existence of potential super-spreaders (Lipschutz-Powell et al. 2013; Anacleto et al. 2015). This is also in line with existing evidence that disease traits are often skewed, and log-transformations are commonly used to satisfy the normality assumptions implied in standard quantitative genetic models. For consistency, the same distributional assumptions were made for susceptibility and infectivity. In particular, polygenic genetic variation was assumed in susceptibility and infectivity, to represent complex traits that are controlled by a large number of genes of small individual effect (Goddard and Hayes 2009).

Thus, the log-transformed susceptibility of offspring *i*, was modelled as

1 $$\log \left( {g_i} \right) = \mu _g + A_{g_i} + e_{g_i}$$

where *μ*_*g*_ was the population mean, $A_{g_i}$ was the additive genetic value for susceptibility, and the environmental effect was drawn from a normal distribution $e_{g_i}\sim N\left( {0,\sigma _{Eg}^2} \right)$ with environmental variance $\sigma _{Eg}^2$ (Table 1). Similarly, the log-transformed infectivity of offspring *i* was modelled as

2 $$\log \left( {f_i} \right) = \mu _f + A_{f_i} + e_{f_i}$$

where *μ*_*f*_ was the population mean, $A_{f_i}$ was the true additive genetic value for infectivity and $e_{f_i}\sim N(0,\sigma _{Ef}^2)$ (Table 1).

**Table 1:** Simulation scenarios

Parameter	Basic parameter values	Alternative parameter values
*N, n*_sires_, *n*_damspersire,_ *n*_offspring per sire/dam_	10,000, 200, 50, 1	–
*t*_generations of selection per replicate_, *n*_replicates_	20, 50	–
Genetic variance for susceptibility in baseline population $({\boldsymbol{\sigma }}_{{\boldsymbol{Ag}}}^2)$	0.5	0.2
Genetic variance for infectivity in baseline population $({\boldsymbol{\sigma }}_{{\boldsymbol{Af}}}^2)$	0.5	0.2
Environmental variance for susceptibility $({\boldsymbol{\sigma }}_{{\boldsymbol{Eg}}}^2)$	2	0.8
Environmental variance for infectivity $({\boldsymbol{\sigma }}_{{\boldsymbol{Ef}}}^2)$	2	0.8
Average effective contact rate (*β*)	0.02	–
Recovery rate (*γ*)	0.017	
Selection accuracy for susceptibility (*r*_*g*_)	0.7	NA, 0.7
Selection accuracy for infectivity (*r*_*f*_)	NA	0.7, 0.5, 0.4, 0.3, 0.2
Selected proportions of sires for susceptibility	0.5	NA, 0.5
Selected proportions of sires for infectivity	NA	0.5, 0.8
Selected proportions of dams for susceptibility	1	–
Selected proportions of dams for infectivity	1	–
Contemporary group size	100	20

Basic and alternative parameter values assumed in the simulation scenarios. NA corresponds to selection only on susceptibility or only on infectivity

The additive genetic effects $A_{g_i}$ and $A_{f_i}$ for offspring *i* were calculated from the average true breeding value of its sire and dam, for susceptibility and infectivity as follows:

3 $$A_{g_i} = ({\mathrm {TBV}}_{{\mathrm {sire}}_{g_i}} + {\mathrm {TBV}}_{{\mathrm {dam}}_{g_i}})/2 + {\mathrm {MS}}_{g_i}$$

4 $$A_{f_i} = ({\mathrm {TBV}}_{{\mathrm {sire}}_{f_i}} + {\mathrm {TBV}}_{{\mathrm {dam}}_{f_i}})/2 + {\mathrm {MS}}_{f_i}$$

where the sire and dam true breeding values for susceptibility (${\mathrm {TBV}}_{{\mathrm {sire}}_g}$ and ${\mathrm {TBV}}_{{\mathrm {dam}}_g}$) and infectivity (${\mathrm {TBV}}_{{\mathrm {sire}}_f}$ and ${\mathrm {TBV}}_{{\mathrm {dam}}_f}$) were simulated from normal distributions with mean zero and additive genetic variance for susceptibility $\sigma _{Ag}^2$, and for infectivity $\sigma _{Af}^2$ (defined in the sections “Generating the base population” and “Updating susceptibility and infectivity trait values”), and ${\mathrm {MS}}_{g_i}$ and ${\mathrm {MS}}_{f_i}$ were the Mendelian segregation terms of individual *i* sampled from normal distributions $N\left( {0,\sigma _{Ag}^2/2} \right)$ for susceptibility, and $N\left( {0,\sigma _{Af}^2/2} \right)$ for infectivity, reflecting the random sampling of parental alleles. Unless stated otherwise, susceptibility and infectivity were assumed to have genetic and environmental correlations of zero.

#### Generating the base population

In the initial population true breeding values for offspring *i* were calculated from Eqs. (3) and (4). Sire and dam true breeding values were distributed as $N\left( {0,\sigma _{Ag}^2} \right)$ and $N\left( {0,\sigma _{Af}^2} \right)$, where $\sigma _{Ag}^2$ and $\sigma _{Af}^2$ were the assumed additive genetic variances for susceptibility and infectivity (Table 1). Then the log-transformed susceptibility and infectivity of offspring *i* of the base generation were calculated from Eqs. (1) and (2), where $\mu _{g_{{\mathrm {base}}}} = 0$ and $\mu _{f_{{\mathrm {base}}}} = 0$, and with environmental effects $e_{g_i}\sim N\left( {0,\sigma _{Eg}^2} \right)$ and $e_{f_i}\sim N\left( {0,\sigma _{Ef}^2} \right)$, where $\sigma _{Eg}^2$ and $\sigma _{Ef}^2$ were the assumed environmental variances for susceptibility and infectivity respectively (Table 1).

#### Calculating response to genetic selection in susceptibility and infectivity

Genetic selection alters trait means and variances over subsequent generations (Falconer and Mackay 1996). Response to selection in susceptibility and infectivity, and change of corresponding population means and genetic variances of these traits, were calculated over 20 discrete generations, following standard quantitative genetic theory as outlined below.

##### Change of trait means

Response to selection per generation assuming discrete generations, was predicted from *R*_*g*_ = *i*_*g*_*r*_*g*_*σ*_*Ag*_ for susceptibility, and *R*_*f*_ = *i*_*f*_*r*_*f*_*σ*_*Af*_ for infectivity (Falconer and Mackay 1996), where *r*_*g*_ and *r*_*f*_ were the assumed selection accuracies (Table 1). The intensity of selection, assuming selection only on the sires, was calculated as $i_g = 1/2\,i_{g_{{\mathrm {sires}}}} + 1/2\,i_{g_{{\mathrm {dams}}}}$ for susceptibility, and $i_f = 1/2\,i_{f_{{\mathrm {sires}}}} + 1/2\,i_{f_{{\mathrm {dams}}}}$ for infectivity, with the magnitude of $i_{g_{{\mathrm {sires}}}}$ and $i_{f_{{\mathrm {sires}}}}$ corresponding to the proportion of selected sires for susceptibility and infectivity respectively (Table 1), and with $i_{g_{{\mathrm {dams}}}} = i_{f_{{\mathrm {dams}}}} = 0$. For each generation, the genetic standard deviations of the log-transformed susceptibility *σ*_*Ag*_ and infectivity *σ*_*Af*_ were calculated as described in the section “Change of trait varainces”.

At generation *k*, the population means for the log-transformed susceptibility and infectivity respectively were:

$$\mu _{g_k} = \mu _{g_{k - 1}} + R_g,\,{\mathrm{and}}\,\mu _{f_k} = \mu _{f_{k - 1}} + R_f$$

where $\mu _{g_k}$ and $\mu _{f_k}$ at generation *k* were less than $\mu _{g_{k - 1}}$ and $\mu _{f_{k - 1}}$ at generation *k* − 1.

##### Change of trait variances

Assuming selection only on the sires, as is common practice in livestock breeding programmes, the sire additive genetic variance for susceptibility at generation *k* was calculated as $\sigma _{Ag_{{\mathrm {sire}}_k}}^2 = \left( {1 - r_g^2k_g} \right)(1/4)\sigma _{Ag_{k - 1}}^2$ where $k_g = (1/2)k_{g_{{\mathrm {sires}}}} + \ (1/2)k_{g_{{\mathrm {dams}}}}$, with $k_{g_{{\mathrm {sires}}}} = i_{g_{{\mathrm {sires}}}}(i_{g_{{\mathrm {sires}}}} - x_{g_{{\mathrm {sires}}}})$ and *k*_dams_ = 0 (Bulmer 1971); $x_{g_{{\mathrm {sires}}}}$ was the deviation of the truncation point from the mean for selection on the sires, in standard deviation units (Falconer and Mackay 1996). Similarly for infectivity, the sire additive genetic variance after selection at generation *k* was $\sigma _{Af_{{\mathrm {sire}}_k}}^2 = \left( {1 - r_f^2k_f} \right)(1/4)\sigma _{Af_{k - 1}}^2$.

Offspring genetic variance $\sigma _{Ag_k}^2$ for susceptibility at generation *k* was calculated from the sire additive genetic variance $\sigma _{Ag_{{\mathrm {sire}}_k}}^2$, the dam additive genetic variance which, assuming no selection on the dams it was equal to the population additive genetic variance $\sigma _{Ag}^2$ in generation *k −* 1, and, the Mendelian sampling variance, which assuming an infinitesimal genetic model and a large breeding population, was equal to the assumed additive genetic variance for susceptibility $\sigma _{Ag}^2$ in the base population (Table 1) (Van Der Waaij et al. 2000):

$$\sigma _{Ag_k}^2 = \left( {1/4} \right)\sigma _{Ag_{{\mathrm {sire}}_k}}^2 + \left( {1/4} \right)\sigma _{Ag_{k - 1}}^2 + \left( {1/2} \right)\sigma _{Ag}^2$$

Similarly, the offspring genetic variance $\sigma _{Af_k}^2$ for infectivity at generation *k* was calculated as

$$\sigma _{Af_k}^2 = \left( {1/4} \right)\sigma _{Af_{{\mathrm {sire}}_k}}^2 + \left( {1/4} \right)\sigma _{Af_{k - 1}}^2 + \left( {1/2} \right)\sigma _{Af}^2$$

#### Updating susceptibility and infectivity trait values

For subsequent generations, offspring true breeding values were calculated using Eqs. (3) and (4) with $A_{g_{ik}}\sim N\left( {0,\sigma _{Ag_k}^2} \right)$ for susceptibility, and $A_{f_{ik}}\sim N\left( {0,\sigma _{Af_k}^2} \right)$ for infectivity, where $\sigma _{Ag_k}^2$ and $\sigma _{Af_k}^2$ were the updated, i.e. post-selection, offspring genetic variances for susceptibility and infectivity at generation *k*, calculated in the section “Change of trait variances”. Log-transformed susceptibility log(*g*_*i*_)_*k*_ and infectivity log(*f*_*i*_)_*k*_ phenotypes at generation *k* were calculated from Eqs. (1) and (2), but using the updated offspring true breeding values $A_{g_{ik}}$ and $A_{f_{ik}}$, and using the updated trait means $\mu _{g_k}$ and $\mu _{g_k}$ for generation *k* from the section “Change of trait means”.

### Modelling the epidemiological impact of selection on susceptibility and infectivity

#### Within-group epidemic transmission model incorporating genetic variation in susceptibility and infectivity

To extract measures of epidemic risk and severity, for every generation, the populations under selection from the section “Modelling genetic selection for lower susceptibility and infectivity” were simulated to be undergoing epidemics as follows:

In each generation *k*, individuals defined by their susceptibility and infectivity phenotypes ((*g*_*i*_)_*k*_ and (*f*_*i*_)_*k*_) generated in the section “Updating susceptibility and infectivity trait values”, were randomly distributed into 100 groups of the same size. The group was the epidemiological unit in which individuals were in direct or indirect (e.g. through sharing the same infectious environment) contact with each other, e.g. management groups in cattle or sheep herds, buildings for broilers in poultry farms, pig pens, or fish-tanks and ponds in aquaculture. Within each group, infection was introduced by one randomly chosen infected and infectious individual; i.e. the index case. Epidemics were simulated within groups, so that each group was a distinct, closed unit, where no between-group transmission occurred.

Representative of a large range of diseases, each epidemic was simulated as a stochastic compartmental Susceptible-Infected-Recovered (SIR) model to provide predictions of epidemic risk and severity. In this model individuals could progress between three states: ‘Susceptible’ where individuals were not infected but were susceptible to infection, ‘Infected’ where individuals were infected and infectious, and ‘Recovered’ where individuals had recovered from infection. Except for the index cases, all individuals at the beginning of an epidemic were considered to be ‘Susceptible’. As no demography was assumed (i.e. no birth, migration or death), each epidemic was simulated until there were no remaining infected individuals in that group.

Epidemics were modelled as stochastic processes in which the number of ‘Susceptible’, ‘Infected’ and ‘Recovered’ individuals changed over time, depending on two types of transition events that could occur: infection of a susceptible individual, or recovery of an infected individual. Individual susceptibility and infectivity affected the infection events in the epidemiological model as follows: infection was modelled as a Poisson process, where an individual’s infection rate depended on the susceptibility of the focal individual and the infectivity of infected group members (Anacleto et al. 2015). The time-varying individual infection rate *λ*_*j*_(*t*) of individual *j* in a group containing *n*(*t*) infected individuals at time *t* was modelled as

5 $$\lambda _j\left( t \right) = g_j\beta \mathop {\sum }\limits_{i = 1}^{n(t)} f_i$$

where *β* was the average effective contact rate (i.e. the assumed rate of contacts between susceptible and infected individuals resulting in infection), and *g*_*j*_ and *f*_*i*_ were the susceptibility phenotype of individual *j* and the infectivity phenotype of individual *i* respectively, generated in the section “Updating susceptibility and infectivity trait values”, where the sum was over all group mates of individual *j* that were infected at time *t* (Anacleto et al. 2015). The above equation for the infection rate demonstrates that reduction in individual susceptibility or infectivity reduces individuals’ infection rates and hence the incidence of infection in the population.

According to this formulation, susceptibility and infectivity are modelled as individual deviations from the average effective contact rate *β*. In homogeneous populations, *g*_*j*_ and *f*_*i*_ are set to unity, to produce the classical expression of the density-dependent force of infection *λ*(*t*) = *βI*(*t*), where *I*(*t*) is the number of infected individuals at time *t* (Keeling and Rohani 2008). Recovery events were assumed to follow an exponential distribution with equal recovery rate *γ* for all individuals (Table 1). Simulations of the epidemics comprised calculations of inter-event times, corresponding event types, and individual experiencing the transition to the next SIR compartment. Those were performed using Gillespie’s direct algorithm (Gillespie 1977), as outlined in more detail in (Lipschutz-Powell et al. 2012a; Anacleto et al. 2015).

#### Assessing the impact of selection on epidemic characteristics

##### Qualitative assessment of SIR profiles

SIR profiles that show proportions of susceptible, infected and recovered individuals during the course of the epidemics, were produced to qualitatively assess the changes in the profiles of the generated epidemics due to selection. The generated profiles over generations were compared between different selection schemes including either susceptibility alone or both susceptibility and infectivity.

##### Epidemic risk

Epidemic risk was assessed over subsequent generations of selection through the basic reproductive ratio *R*_0_, and through the proportion of epidemics that occurred.

Firstly, estimates of changes in the basic reproductive ratio *R*_0_ over generations of selection were obtained through stochastic simulations. In those, epidemics were simulated following the SIR model as described in the section “Within-group epidemic transmission model incorporating genetic variation in susceptibility and infectivity”, but with the difference that in each group only the index case was allowed to infect other susceptible individuals. This produced a simulated *R*_0_ for every group. Summary *R*_0_ values per generation were obtained by calculating the mean and the median of the simulated *R*_0_ values across groups for each generation. Although the mean *R*_0_ is more in line with the classical definition of *R*_0_ (e.g. Diekmann et al. 1990), the distribution of *R*_0_ values over groups was skewed due to the random allocation of individuals with different (skewed) susceptibility and infectivity values into groups. Hence the median over groups of the simulated *R*_0_ values, termed herein ‘realised’ *R*_0_, was used as a more appropriate summary statistic in the present analysis. Finally, the overall mean of the summary *R*_0_ statistic per generation with standard errors were calculated over 50 replicates. The overall trends of decline over generations of selection for the mean *R*_0_ were similar to those for the median (Supplementary Information 1).

Secondly, the mean proportion of epidemics that occurred, i.e. the proportion of epidemics where the index case generated secondary cases, was calculated for each generation. This allowed to explore how often epidemics with *R*_0_ ≥ 1 occur. Presented values are the means with standard errors over 50 replicates.

##### Epidemic severity

For the groups where epidemics occurred, i.e. where the index cases generated secondary cases, epidemic severity was assessed over subsequent generations of selection through the proportion of infected animals, and through the epidemic duration.

Firstly, the mean proportion of infected individuals was calculated across the groups with at least one secondary case. Means and standard errors were obtained over 50 replicates, after excluding replicates where all groups had only the index case infected.

Secondly, the epidemic duration was calculated for each group as the time-point when there were no remaining infected individuals in the group. The 33% and 66% percentiles of the duration of epidemics in the base generation, after excluding groups where no epidemics occurred, were used to classify epidemics as ‘short’, ‘medium’ and ‘long’. The changes in the mean proportions of ‘no’, ‘short’, ‘medium’ and ‘long’ epidemics with standard errors were assessed over generations.

### Simulation scenarios and investigation of parameter space

Parameters were chosen to represent population structures and routine genetic evaluations realistic for the livestock industry, and disease epidemics that may emerge within livestock production systems, so that both assumed and predicted values were realistic for those systems, as follows:

Genetic variance in susceptibility and infectivity is a driving parameter for the genetic gain that can be achieved in those traits from selection. Furthermore, genetic variance in susceptibility and infectivity affects epidemic risk and severity through its effect on the infection rate (see section “Within-group epidemic transmission model incorporating genetic variation in susceptibility and infectivity”), hence driving the resulting *R*_0_ (see section “Epidemic risk”). Therefore, different levels of genetic and environmental variation were simulated, such that the resulting R_0_ values represented mild and severe epidemics, respectively (see section “Epidemic risk”): $\sigma _{Ag}^2 = \sigma _{Af}^2 = 0.5$ and $\sigma _{Eg}^2 = \sigma _{Ef}^2 = 2$; and, $\sigma _{Ag}^2 = \sigma _{Af}^2 = 0.2$ and $\sigma _{Eg}^2 = \sigma _{Ef}^2 = 0.8$ (Table 1). Those values covered a broad range of genetic variances, and a realistic spectrum of disease *R*_0_ by focusing on mild epidemics with *R*_0_ in the region of 2 (e.g. Charpin et al. 2012), or severe epidemics with *R*_0_ in the region of 7 (e.g. Le Menach et al. 2005). The effect on the infection rate, and hence on the epidemic, of the genetic variance in susceptibility and infectivity, is confounded with the effect of the average effective contact rate *β* (see section “Within-group epidemic transmission model incorporatinggenetic variation in susceptibility and infectivity”), hence *β* was fixed to 0.02 and the variances were allowed to vary (Table 1). The above combination of variances for susceptibility and infectivity allowed to assume heritabilities of 0.2 for both latent traits (defined on the underlying scale), which corresponds to a lower heritability on the observed scale (i.e. binary infection status) within the range reported for common diseases (Brotherstone et al. 2010; Kemper et al. 2011; Boddicker et al. 2012; Bermingham et al. 2014a; Tsairidou et al. 2014; Raphaka 2018). In addition, previous studies have shown that heritability is likely to be underestimated in the context of infectious diseases due to incomplete exposure to infection (Bishop and Woolliams 2010). Hence, the heritability of 0.2 presented here, is a realistic and rather conservative value.

The recovery rate in the epidemiological SIR model was calculated as the reciprocal of the infectious period (Keeling and Rohani 2008) which was assumed ~2 months, corresponding either to the true infectious period, e.g. of a viral infection, such as PRRS in pigs (Nodelijk et al. 2000), or to the diagnostic testing intervals in an eradiation scheme, e.g. the 60-day interval for bovine Tuberculosis testing in GB (De La Rua-Domenech et al. 2006).

A range of selection accuracies was simulated for infectivity, given an optimal but realistic accuracy of *r*_*g*_ = 0.7 for susceptibility, which corresponds to a reliability for sire EBVs of 0.5 as reported in Banos et al. (2017) and as expected from genomic technologies and sequencing data given sufficiently large populations. In contrast, emerging evidence from simulation studies suggests that infectivity is a trait more challenging to measure accurately (Anacleto et al. 2015; Anche et al. 2015; Biemans et al. 2017), and therefore, we evaluated accuracies for infectivity ranging from *r*_*f*_ = 0.2–0.7 (Table 1).

Genetic gain achieved through selection is affected by the applied selection intensity, therefore, the following proportions of selected sires for susceptibility and infectivity were simulated (Table 1): 50% selection for susceptibility which corresponds to selection intensity of *i*_*m*_ = 0.798, but no selection for infectivity; 50% selection for infectivity, but no selection for susceptibility; 50% selection for both infectivity and susceptibility; 50% selection for susceptibility and 80% selection for infectivity corresponding to lower selection intensity *i*_*m*_ = 0.35 for infectivity. The latter scenario represents a moderate selection scheme regarding infectivity, that would exploit infectivity only to identify and remove designated super-spreaders.

Finally, alternative contemporary group sizes were simulated keeping the population size constant (Table 1).

## Results

### SIR profiles

**Fig. 2:**
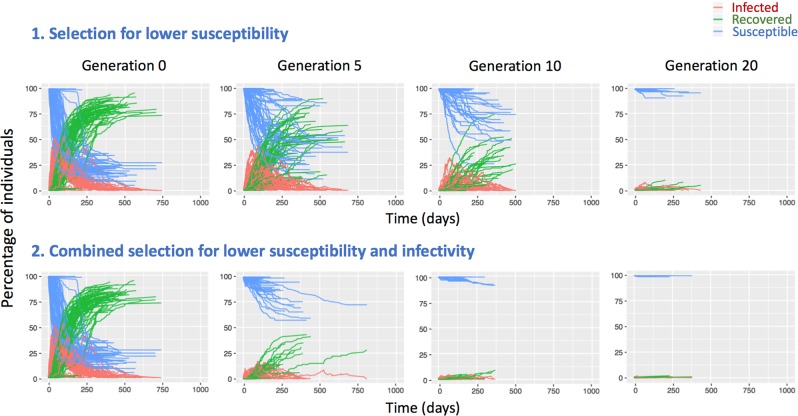
SIR profiles for the genetic variances of 0.5. Example from one replicate, of the SIR profiles for genetic variance of 0.5, over generations of selection: (**a**) only on susceptibility (upper row) and (**b**) on both susceptibility and infectivity (lower row), with selection accuracies of 0.7

Selection for susceptibility alone reduced the number and severity of epidemics occurring, over generations (Fig. 2). However, this decline was more prominent and required fewer generations, when selection was on susceptibility and infectivity combined. In addition, combined selection generated a stronger and quicker decline in the epidemic severity in most individual epidemics, with a quicker elimination of longer epidemics compared to selection on susceptibility alone (Fig. 2). These effects were observed for both larger ($\sigma _{Ag}^2 = \sigma _{Af}^2 = 0.5$) and smaller ($\sigma _{Ag}^2 = \sigma _{Af}^2 = 0.2$) genetic variances simulated (results for genetic variance of 0.2 are shown in Supplementary Information 2).

### Epidemic risk

**Fig. 3:**
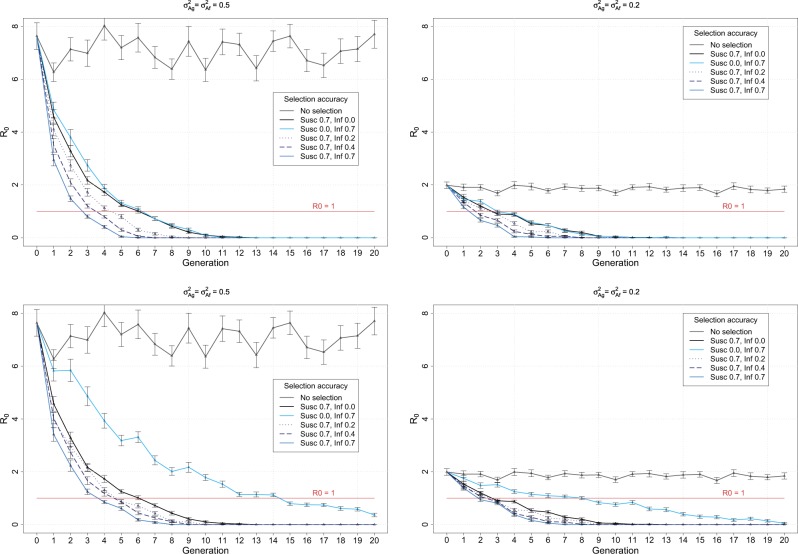
Change in the population realised *R*_0_ over generations of selection. The graphs show the change per generation in the population realised *R*_0_ for different genetic variances and selection intensities (Table 1). Upper row: 50% selection on sires for both susceptibility and infectivity, for the genetic variances of 0.5 (left panel) and 0.2 (right panel). Lower row: 50% selection on the sires for susceptibility and 80% selection on the sires for infectivity, for the genetic variances of 0.5 (left panel) and 0.2 (right panel). The vertical bars represent the standard errors over 50 replicates. The red line shows the *R*_0_ threshold value of 1

Response to genetic selection in susceptibility and infectivity generated a profile of decline in the mean population susceptibility and infectivity over generations which was faster within earlier generations, as expected from selection using the assumed parameter values (see Supplementary Information 3). In the base generation before selection, the mean realised *R*_0_ over replicates was 7.64 when assuming a genetic variance of 0.5, and 1.99 for a genetic variance of 0.2. Over generations, realised *R*_0_ declined due to genetic selection. The selection scheme combining susceptibility and infectivity, considerably reduced the number of generations required until realised *R*_0_ was <1; i.e. until disease eradication, compared to selection on susceptibility alone (Fig. 3). Specifically for the genetic variance of 0.5, selection on susceptibility required seven generations to bring the realised *R*_0_ from 7.64 to a value below 1 (Fig. 3). In contrast, combined selection, with the same selection intensities and accuracies for susceptibility and infectivity, required three generations to bring the realised *R*_0_ below 1 (Fig. 3). Selecting on infectivity alone produced similar changes in epidemic risk as selecting on susceptibility alone (Fig.3). For example, both 50% selection on susceptibility alone, or 50% selection on infectivity alone, reduced the realised *R*_0_ below 1 after seven generations of selection (Fig.3). For lower genetic variance of 0.2 resulting in lower realised *R*_0_ in the base generation, fewer generations were generally required until disease eradication, although the actual rate of decline in *R*_0_ was lower compared to that corresponding to larger genetic variance. For genetic variance of 0.2, there was a one-generation difference between selection schemes considering susceptibility or infectivity alone and combined selection including infectivity (Fig. 3).

When applying less stringent selection intensity for infectivity (80% selection for infectivity corresponding to removing only designated super-spreaders) and for the genetic variance of 0.5, selection on both susceptibility and infectivity with selection accuracies of 0.7 required four generations to reduce the realised *R*_0_ below 1, compared to three generations required with higher selection intensity. The biggest difference was observed for selection on infectivity alone, which, with only 80% selection, required 15 generations to reduce the realised *R*_0_ below 1 (Fig. 3). Even with this less stringent selection scheme for infectivity, incorporating infectivity in addition to susceptibility achieved disease eradication within four generations compared to seven generations required for selection on susceptibility alone (Fig. 3).

**Fig. 4:**
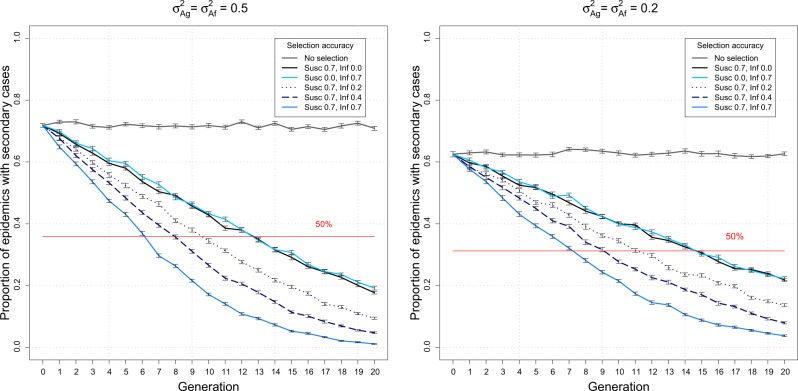
Proportion of epidemics with at least one secondary case over generations of selection. The graphs show the proportion of groups that resulted in at least one secondary case after introduction of an arbitrary infected index case, assuming a genetic variance of 0.5 (left panel), or 0.2 (right panel) (Table 1). The vertical bars represent the standard errors of the means over 50 replicates. The red line denotes the 50% benchmark

The change in epidemic risk due to selection was also assessed by examining the proportion of groups that resulted in an epidemic with at least one secondary case generated by the index case. In the base generation, for genetic variance of 0.5, on average 72% of groups had at least one secondary case (Fig. 4). After seven generations of combined selection for susceptibility and infectivity, the proportion of epidemics occurring was reduced by at least 50%, while selection on susceptibility alone required 13 generations to achieve the same outcome (Fig. 4). It required 15 generations of combined selection to reduce epidemic risk below 5%, whereas with selection only on susceptibility or only on infectivity, it was not possible to achieve this result within the 20 generation duration of the selection scheme. Even with lower selection accuracies for infectivity (e.g. 0.2), a significant reduction of disease risk was achieved with combined selection (Fig. 4). Similar patterns were observed for the genetic variance of 0.2, where combined selection with accuracy of 0.7 for both susceptibility and infectivity generated a significantly greater reduction in the proportion of epidemics occurring compared to selection on susceptibility alone (Fig. 4).

### Epidemic severity

**Fig. 5:**
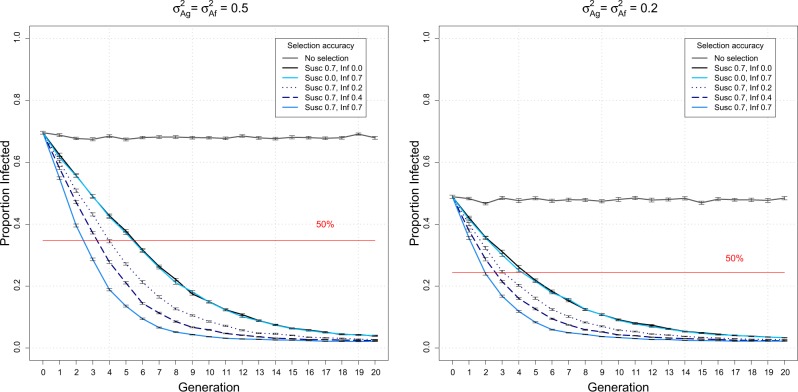
Epidemic severity over generations of selection. The graphs show the proportion of infected individuals across generations in the groups where there were secondary cases, assuming a genetic variance of 0.5 (left panel), or 0.2 (right panel) (Table 1). The vertical bars represent the standard errors of the means over 50 replicates. The red line denotes the 50% benchmark

In the epidemics that occurred, genetic selection also produced a non-linear reduction in the proportion of infected individuals, such that the greatest benefit was observed within the first few generations. (Fig. 5). For genetic variance of 0.5, after three generations of combined selection for susceptibility and infectivity with accuracies of 0.7 for both traits, the average proportion of infected individuals was reduced by at least 50%. It required six generations of selection on susceptibility alone, or on infectivity alone, to reduce this proportion by the same amount. Even when selection accuracy for infectivity was low (e.g. 0.2), there was a significant improvement with combined selection compared to selection on susceptibility, or on infectivity alone (Fig. 5). For smaller genetic variance of 0.2, the average proportion of infected individuals was lower, and differences between selection scenarios with respect to different selection accuracies, were less pronounced (Fig. 5). Nevertheless, two generations of combined selection were sufficient for reducing the proportion of infected individuals by at least 50%, while selection on susceptibility alone, or infectivity alone, required five generations.

Selection reduced the duration of epidemics, with no long epidemics observed after 10 generations of combined selection. Notably, selection on susceptibility alone required 20 generations to achieve the same outcome (Table 2). Similar trends were observed in results for the genetic variance of 0.2, and those are shown in Supplementary Information 4.

**Table 2:** Proportion of short and long epidemics over generations of selection, for the genetic variance of 0.5

Generation	Selection on susceptibility						Selection on susceptibility and infectivity					
	No epidemic	se	Short	se	Long	se	No epidemic	se	Short	se	Long	se
0	0.28	0.01	0.24	0.01	0.24	0.01	0.28	0.01	0.24	0.01	0.24	0.01
1	0.31	0.01	0.24	0.01	0.24	0.01	0.35	0.01	0.24	0.01	0.23	<10−^2^
2	0.34	0.01	0.26	0.01	0.23	0.01	0.41	0.01	0.28	0.01	0.18	0.01
3	0.37	0.01	0.25	0.01	0.22	0.01	0.46	0.01	0.30	0.01	0.14	<10^−2^
4	0.40	0.01	0.27	0.01	0.20	0.01	0.53	0.01	0.32	0.01	0.08	<10^−2^
5	0.42	0.01	0.28	0.01	0.18	0.01	0.57	0.01	0.33	0.01	0.06	<10^−2^
6	0.46	0.01	0.29	0.01	0.15	<10^−2^	0.63	0.01	0.31	0.01	0.03	<10^−2^
7	0.50	0.01	0.30	0.01	0.11	0.01	0.70	0.01	0.26	0.01	0.02	<10^−2^
8	0.51	0.01	0.31	0.01	0.10	<10^−2^	0.74	0.01	0.24	0.01	0.01	<10^−2^
9	0.54	0.01	0.32	0.01	0.08	<10^−2^	0.78	<10^−2^	0.20	<10^−2^	0.01	<10^−2^
10	0.57	0.01	0.31	0.01	0.06	<10^−2^	0.83	<10^−2^	0.16	<10^−2^	0.00	<10^−2^
11	0.61	0.01	0.30	0.01	0.04	<10^−2^	0.86	0.01	0.13	<10^−2^	0.00	<10^−2^
12	0.62	0.01	0.31	0.01	0.04	<10^−2^	0.89	<10^−2^	0.10	<10^−2^	0.00	<10^−2^
13	0.65	0.01	0.29	0.01	0.03	<10^−2^	0.91	<10^−2^	0.09	<10^−2^	0.00	<10^−2^
14	0.68	0.01	0.27	0.01	0.02	<10^−2^	0.93	<10^−2^	0.07	<10^−2^	0.00	<10^−2^
15	0.71	0.01	0.26	0.01	0.01	<10^−2^	0.95	<10^−2^	0.05	<10^−2^	0.00	<10^−2^
16	0.74	0.01	0.23	0.01	0.01	<10^−2^	0.95	<10^−2^	0.04	<10^−2^	0.00	<10^−2^
17	0.76	0.01	0.22	0.01	0.01	<10^−2^	0.97	<10^−2^	0.03	<10^−2^	0.00	<10^−2^
18	0.77	0.01	0.21	0.01	0.01	<10^−2^	0.98	<10^−2^	0.02	<10^−2^	0.00	<10^−2^
19	0.80	<10^−2^	0.19	<10^−2^	0.01	<10^−2^	0.98	<10^−2^	0.02	<10^−2^	0.00	<10^−2^
20	0.82	<10^−2^	0.17	<10^−2^	0.00	<10^−2^	0.99	<10^−2^	0.01	<10^−2^	0.00	<10^−2^

Proportions of ‘short’, ‘medium’ and ‘long’ epidemics classified on the basis of the 33% and 66% percentiles of the duration of epidemics in the base generation, for the scenarios of selection on susceptibility alone (with accuracy 0.7), and selection on both susceptbility and infectivity (with accuracies 0.7)

## Discussion

Epidemiological models have proven a powerful tool to inform national and international disease control programmes (Kao 2002; Keeling 2005; Feng et al. 2007; Birch et al. 2018). However, despite the increasing recognition of the vast potential of genomic approaches (Ibanez-Escriche and Simianer 2016; 2018), these models rarely incorporate genetic disease control. Nevertheless, effective implementation of genomic selection for increased host resistance to infectious diseases is increasingly becoming one of the main goals in modern breeding programmes in most domestic livestock and aquaculture species. One of the major drawbacks of genetic disease control compared to non-genetic strategies is that genetics operate on a much slower time-scale, since the benefits of selection only accumulate over successive generations. In order for genetic strategies to offer a viable contribution to disease control and eradication programmes, within the typically short timeframes that such programmes operate on, a rapid reduction in disease incidence as a result of selection is vital. Selection on host susceptibility alone may not achieve this desired outcome (Man et al. 2009; Raphaka et al. 2018). Recent advances in statistical genetics now facilitate estimation of genetic parameters associated with a second host trait affecting disease transmission, i.e. host infectivity (Anacleto et al. 2015; Biemans et al. 2017). However, implementation of this new trait into disease control programmes requires improved disease monitoring schemes and experimental designs for data collection, and it would also affect estimation of economic costs (Janssen et al. 2018). Hence, the impact on disease incidence and epidemic risk that result from including this novel disease trait into genetic selection need to be systematically assessed. However, to date an adequate tool to predict the epidemiological effects of adding infectivity into genetic selection, and how those depend on prediction accuracy and selection intensity as the driving parameters for genetic progress, is lacking. In this study, we developed a genetic-epidemiological prediction model that quantifies the non-linear impact of selection methods considering genetic susceptibility and infectivity in livestock on dynamic epidemic processes, and assesses their impact in terms of practical measures of epidemic risk and severity used in the field. The code used for the models presented in this paper is available on GitHub (https://github.com/SmaragdaT/GenEpi) and can be adapted to match-specific diseases and population structures. The primary findings demonstrate that the additional benefits from capturing both susceptibility and infectivity can be of practical value even when selection intensity for infectivity is relatively low, and this conclusion is robust over a range of selection accuracies.

All measures of epidemic risk and severity were found to be considerably lower when selecting for infectivity in addition to susceptibility, so that benefits from selection were achieved after fewer generations compared to selection for susceptibility alone. These effects were observed for a range of accuracies, with a substantial reduction of disease severity through combined selection, even when selection accuracy for infectivity was 0.2. As expected, the size of the observed effects was found to depend on the magnitude of the genetic variance assumed, with differences between scenarios being less pronounced for smaller genetic variances. In addition it was found that, depending on the amount of genetic variation in infectivity, a selection scheme could be similarly efficient if targeting infectivity rather than susceptibility. These results show that combining selection for susceptibility and infectivity can, not only be more efficient than selection targeting susceptibility alone, but may also achieve disease eradication in cases where selection only on susceptibility would not be sufficient for achieving eradication within a reasonable time-scale (e.g. Fig. 5).

### Genetic selection modelling approach

Susceptibility to infectious diseases in livestock has commonly been found to be a complex, polygenic trait, for example, in bovine mastitis (Heringstad et al. 2000) and bovine tuberculosis (Bermingham et al. 2014b; Tsairidou et al. 2014), in sea lice infection in Atlantic salmon (Tsai et al. 2016), and in nematode infections (Kemper et al. 2011; Riggio et al. 2013) and footrot (Mucha et al. 2015) in sheep. Hence here, latent susceptibility and infectivity were modelled as quantitative traits, assuming polygenic genetic variation. In the present study, selection was modelled on the latent objective traits: i.e. susceptibility and infectivity, rather than indicator traits based on disease phenotypes. Selection on these latent traits has the benefit that they directly affect individual infection rates and hence disease incidence in the population (Eq. (5)). Uncertainty in the traits was accounted for by simulating a range of selection accuracies.

The selection methodology followed in this study is in line with independent culling levels selection (Tallis 1962; Vinson 1971), which is commonly practiced in cattle and sheep breeding and is effective in excluding animals with particularly poor EBV for a trait of interest. Independent culling may not be the optimum method, and is less efficient than a linear index, when the selection goal is a linear combination of the traits, although this is not the case here.

In the present study, a genetic correlation of zero was assumed between susceptibility and infectivity, while the impact of such correlations was explored in Supplementary Information 5. One might expect a positive phenotypic correlation between susceptibility and infectivity due to the dependency of expressing infectivity upon being susceptible and infected. However, that does not imply the sign and magnitude of the underlying genetic correlation (Rauw et al. 1998). A strong positive genetic correlation would indicate that susceptibility to infection and infectivity are controlled by the same set of genes. However, to the best of the authors’ knowledge, there is no evidence suggesting that whether an individual becomes infected is regulated by the same genetic pathways as those controlling the transmission of infection. Therefore, susceptibility and infectivity should be considered as two genetically separable traits.

As expected, with a favourable genetic correlation between susceptibility and infectivity, combining these traits generated a smaller additional benefit compared to selecting only on susceptibility (Supplementary Information 5; Fig. S5 left panel). This is due to the indirect correlated response arising in infectivity even when selection is on susceptibility alone. A positive correlation implies that individuals with lower susceptibility also tend to have lower infectivity, and selection against these individuals accelerates reduction in disease risk and prevalence. Conversely, when susceptibility and infectivity were antagonistically correlated, there was a substantial delay in the progress achieved by the selection scheme considering only susceptibility. However, considering both susceptibility and infectivity in the selection scheme helped alleviate this delay (Supplementary Information 5; Fig. S5 right panel). Such adverse correlations have been previously observed, for example, between milk yield and fertility traits in dairy cattle (Wall et al. 2003), or in cases of competition over resource allocation (Rauw et al. 1998; Read and Taylor 2001). Therefore, particularly in the case of an unfavourable genetic correlation between susceptibility and infectivity, estimating effects for both these traits can be crucial to avoid an undesirable increase in infectivity, which could counteract or even outweigh the benefits of selection for reduced susceptibility.

### Epidemiological modelling approach

Compartmental Susceptible-Infected-Recovered models have been commonly used to represent the spread of a large class of infections with prolonged immunity (Anderson and May 1992), and the model presented here directly applies to many microparasite infections. This modelling approach can be extended to macroparasite infections, by additionally modelling the infection severity and reproduction cycle of the parasite within the host (Bishop and Stear 1999; Doeschl-Wilson et al. 2008). This model can also be extended to represent infections featuring more compartments, e.g. a ‘Latency’ compartment, or to accommodate genetic variation in more traits, in addition to susceptibility and infectivity, that may also affect the transmission dynamics. Nevertheless, the benefits arising from combined selection on susceptibility and infectivity would still be expected to be substantial.

A further effect observed from the SIR profiles was that although after generations of selection epidemics were generally fewer and milder, in some replicates they were prolonged, ending at a later time-point compared to epidemics in the base generation. In epidemiology it is known that epidemics with lower average transmission coefficient are less severe but can often be prolonged (Keeling and Rohani 2008). Here, the same effect was generated by the change in the infection rate over generations due to selection reducing susceptibility and infectivity.

### Epidemiological group size and family structure

Response to genetic selection for indirect genetic effects, such as infectivity, has been shown to depend on the size of the epidemiological groups (Bijma 2010a; Ødegård and Olesen 2011; Anacleto et al. 2015). Group size is an important factor in planning livestock management practices to optimise disease control, and in designing disease challenge experiments. As shown in Supplementary Information 6, combined selection incorporating both susceptibility and infectivity performs better than selection on susceptibility alone even for a smaller group size. However, the infectivity of early infected individuals (e.g. the randomly chosen index case in our simulations) has a larger influence on disease spread in smaller groups, and thus on the response to selection achieved.

Some studies have suggested that for indirect genetic effects, within-group and between-group genetic covariance, i.e. relatedness, can increase response to selection (Bijma 2010a; Ødegård and Olesen 2011; Anche et al. 2014a). The widespread use of artificial insemination in farmed animal allows popular sires to have a large number of offspring within and across farms. As a consequence, benefits in practice may be higher than for the random allocation assumed here.

The observed response to selection may also be influenced in practice by the reduction of selection accuracy as disease prevalence declines, and by potential changes in the covariance between susceptibility and infectivity that may emerge over generations. Such effects can be minimised by the use of genomic data and large training datasets to reduce the role of phenotypes and maintain the statistical power over generations.

### Implications

By employing classic genetic disease control strategies which only target reduced host susceptibility to disease, it may not be possible to tackle within a reasonable time-scale diseases with large *R*_0_ and diseases with no or very small genetic variance in host susceptibility. In diseases with *R*_0_ much larger than one, selection on susceptibility alone might not be sufficient to reduce *R*_0_ and achieve disease eradication within a reasonable number of generations for a breeding scheme (Mackenzie and Bishop 2001; Bishop et al. 2010). Similarly, the genetic gain achieved would not be of practical value if there was very limited genetic variation in susceptibility available for selection. However, the results of this study reveal that, when subject to heritable variation, infectivity could complement susceptibility to accelerate progress in selection schemes, or could be an alternative disease-related target-trait for genetic disease control. Infectivity as an indirect genetic effect is likely to have been under weaker natural selection pressure, compared to direct genetic effects, therefore there might be more genetic variation in infectivity available for artificial selection compared to susceptibility (Bijma 2010b).

Infection incidence is expected to evolve also due to natural selection and not only due to artificial selection as considered here. The framework of this study could help to investigate the role of evolution in changing genetic variances and covariances in susceptibility and infectivity, which drive evolution in disease dynamics. Evolutionary studies have long recognised that the risk of infection has different components (Elderd et al. 2008), namely: (a) the risk of infection given exposure (which encompasses variation in host susceptibility), and (b) the risk of exposure (which encompasses variation in host infectivity). Furthermore, the Breeder’s equation used in this study to calculate response to selection in host susceptibility and infectivity over successive generations, is commonly used in animal breeding to predict response to artificial selection. However, in studies considering natural selection, it has been previously suggested that the traits of interest might be correlated with other components, which may have not been explicitly accounted for, but which may also affect variation in fitness, and this may have an impact on the response to selection as predicted by the Breeder’s equation (Morrissey et al. 2010). For example, the present simulations do not consider co-evolution between the host and the pathogen, which likely affects infection risk. Cooperation or competition between individuals sharing the same social environment also evolve through natural or artificial selection and that affects the variance in the target traits (Marjanovic et al. 2018). In evolutionary biology terms, selection for lower infectivity may enhance cooperation between the focal individual and its social partners to avoid or survive infection, and hence affect variation in infectivity; for example in plants, selection for less competitive phenotypes has led to more uniform crops (Austin et al. 1980; Denison et al. 2003).

The benefits from selection considering infectivity, in addition to susceptibility, can be particularly strong in the presence of genetic super-spreaders, which would not be captured by selection for susceptibility alone unless this trait is strongly correlated with infectivity. Using genetic information, i.e. infectivity breeding values, super-spreaders could be identified, hence eliminating a major source of infection for other animals. In zoonotic diseases such as bovine Tuberculosis or diseases where environmental contamination influences the risk of infection, such as footrot and nematode infestations in sheep, removing super-spreaders would reduce the shedding of pathogens to wildlife disease vectors or into the environment (Doeschl-Wilson et al. 2011; Tsairidou et al. 2018a).

## Conclusions

In conclusion, host infectivity, in addition to susceptibility, was found to constitute an important trait to target in future genetic and genomic selection schemes to reduce the impact of epidemics in livestock populations more efficiently. Combined selection for reduced susceptibility and infectivity generated a greater reduction in epidemic risk, severity and duration, and with beneficial outcomes of selection emerging earlier, compared to selection targeting susceptibility alone. Advances in genomic technologies and novel statistical methods make it now feasible to determine genetic effects for novel traits that have a substantial impact on infectious disease prevalence but are difficult to measure, such as host infectivity. Therefore, genetic variation in infectivity and its potential benefits for genetic evaluations should be further investigated. The framework proposed here helps predict the impact of artificial selection on future disease dynamics and can facilitate investigation of the role of evolution.

## Supplementary information

Supplementary Information 1

Supplementary Information 2

Supplementary Information 3

Supplementary Information 4

Supplementary Information 5

Supplementary Information 6

## Data Availability

The code used for the analyses presented in this paper can be accessed on GitHub https://github.com/SmaragdaT/GenEpi and is available as an R package (GenEpiSim/R version 3.3.3).
