# Supplementary material for: Enhancing genetic disease control by selecting for lower host infectivity and susceptibility
Source: Heredity (Edinb). 2019 Jan 16;122(6):742–58. doi: 10.1038/s41437-018-0176-9 (PMC6781107; doi:10.1038/s41437-018-0176-9)
Supplement: Supplementary file 1 — Supplementary Information 1 [file 41437_2018_176_MOESM1_ESM.docx]

**Supplementary Information 1**

***Mean values for R_0_***

Decline over generations of the mean realised R_0_ values across groups. Standard errors were calculated over 50 replicates. Overall trends of decline over generations of selection are similar to those for the median of the realised R_0_. The starting realised R_0_ values are larger for the mean (Figure S1) than for the median (Fig. 3) due to the skewed distributions of simulated realised R_0_ values over groups as the results of random sampling of individuals over groups.

**Figure S1. Change in the mean R_0_ values over generations of selection**
