# Supplementary material for: Enhancing genetic disease control by selecting for lower host infectivity and susceptibility
Source: Heredity (Edinb). 2019 Jan 16;122(6):742–58. doi: 10.1038/s41437-018-0176-9 (PMC6781107; doi:10.1038/s41437-018-0176-9)
Supplement: Supplementary file 2 — Supplementary Information 2 [file 41437_2018_176_MOESM2_ESM.docx]

**Supplementary Information 2**

***SIR profiles of epidemics for the genetic variance of 0.2***

By inspecting the SIR profiles of individual epidemics over generations of selection for the smaller genetic variance of 0.2, it can be observed that, similarly to the larger variance simulated (see Results), the number of epidemics occurring and the number of long epidemics decline over generations due to selection. This decline is more pronounced when selection is on both susceptibility and infectivity.

**Figure S2. SIR profiles for the genetic variances of 0.2**

*Example from one replicate, of the SIR curves for genetic variance of 0.2, over generations of selection (a) only on susceptibility (upper row), and (b) on both susceptibility and infectivity (lower row), with selection accuracies of 0.7.* *The grey-shaded areas show the corresponding 95% CI for the fitted curve.*

*
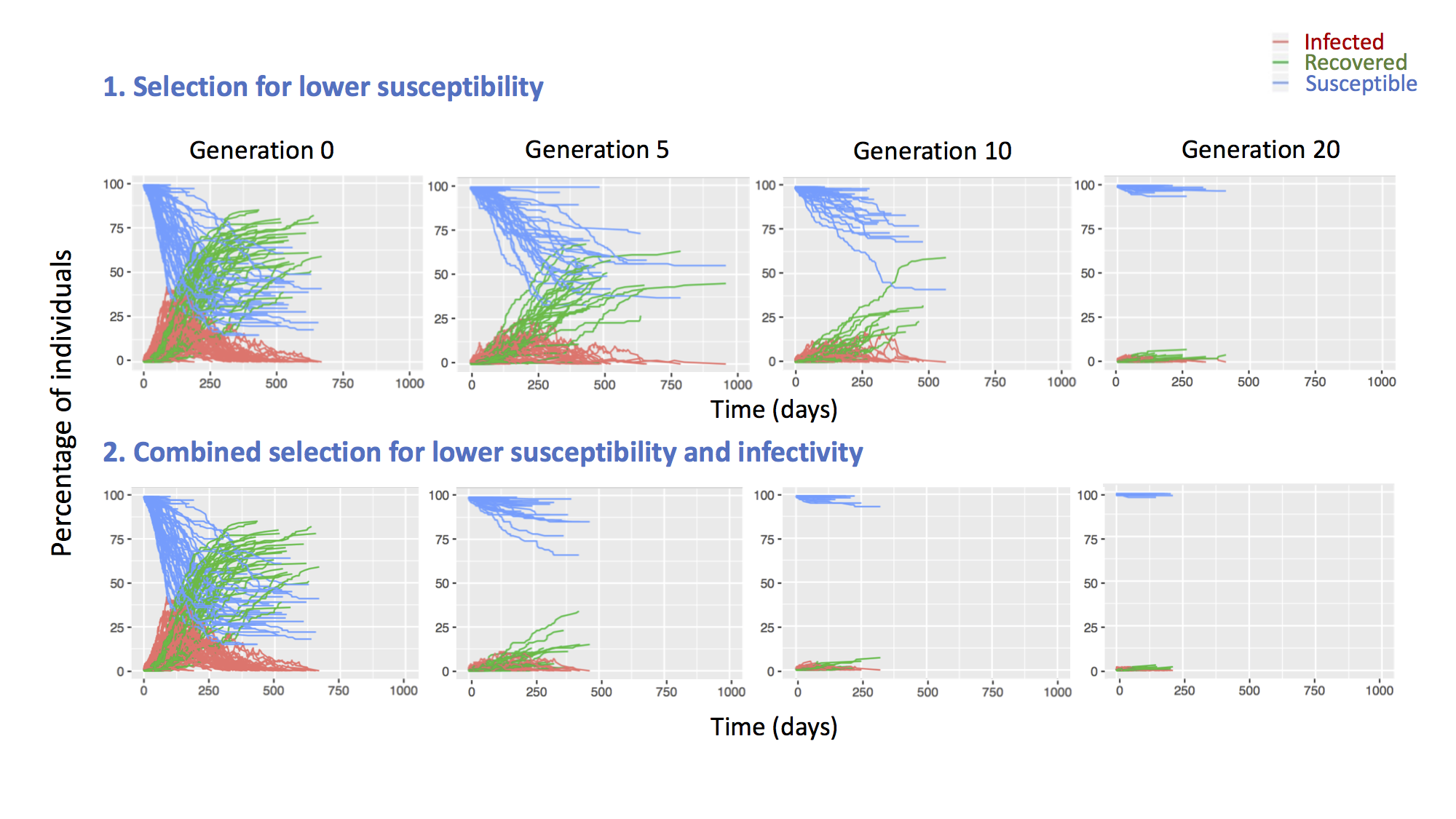
*
