# Supplementary material for: Enhancing genetic disease control by selecting for lower host infectivity and susceptibility
Source: Heredity (Edinb). 2019 Jan 16;122(6):742–58. doi: 10.1038/s41437-018-0176-9 (PMC6781107; doi:10.1038/s41437-018-0176-9)
Supplement: Supplementary file 3 — Supplementary Information 3 [file 41437_2018_176_MOESM3_ESM.docx]

**Supplementary Information 3**

***Impact of selection on the mean simulated population susceptibility and infectivity over generations***

Figure S3 shows the change in mean latent susceptibility and mean latent infectivity on the log-normal scale for different genetic variances and with varying infectivity selection accuracy.

Genetic selection for low susceptibility (infectivity) reduced the mean population susceptibility (infectivity) over generations, with a steeper decline within earlier generations and with a greater reduction in infectivity for higher selection accuracies (Fig. S3).

After 4 generations of selection, susceptibility (infectivity) was reduced by at least 50% for selection accuracy of 0.7, while for selection accuracy of 0.2 infectivity was reduced by at least 50% after 15 generations. Existence of genetic variation in infectivity and selection on this trait did not affect response to selection in susceptibility and vice versa when susceptibility and infectivity were assumed independent.

When the simulated genetic variance for the traits was smaller, there was a slightly smaller reduction in the mean population susceptibility and infectivity due to selection within the same time-frame (Fig. S3). After 7 generations of selection susceptibility (infectivity) was reduced by at least 50% for selection accuracy of 0.7. More than 20 generations of selection were required to reduce infectivity by 50% with selection accuracy of 0.2.

**Figure S3. Change in mean susceptibility and infectivity.**

**
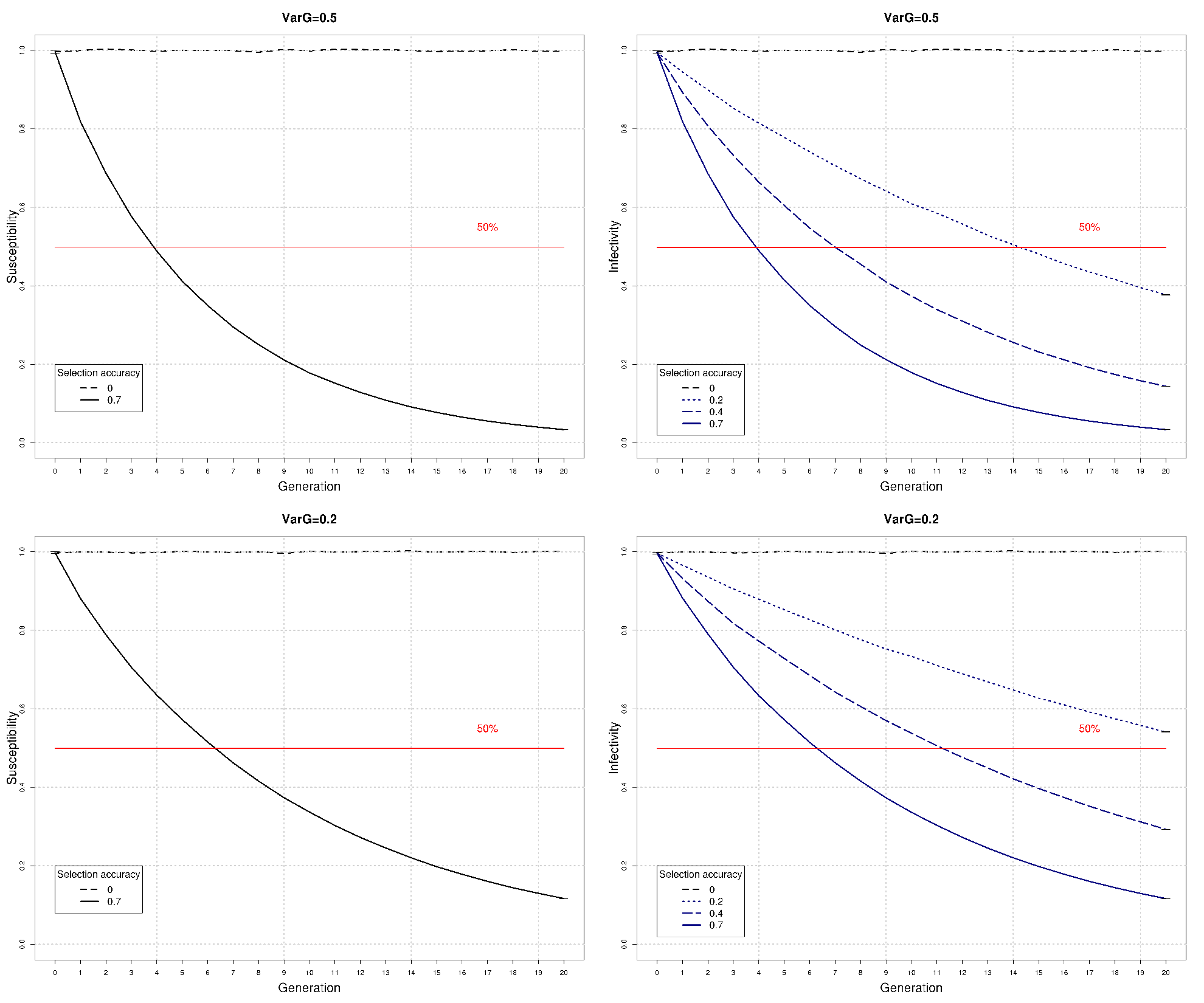
**

*Mean of simulated population susceptibility and infectivity on the log-normal scale over 20 generations of selection for varying selection accuracies with SEs over 50 replicates.*
