# Supplementary material for: Enhancing genetic disease control by selecting for lower host infectivity and susceptibility
Source: Heredity (Edinb). 2019 Jan 16;122(6):742–58. doi: 10.1038/s41437-018-0176-9 (PMC6781107; doi:10.1038/s41437-018-0176-9)
Supplement: Supplementary file 4 — Supplementary Information 4 [file 41437_2018_176_MOESM4_ESM.docx]

**Supplementary Information 4**

***Impact of selection on epidemic duration for the genetic variance of 0.2***

Smaller genetic variance resulted in higher proportion of short epidemics compared to long epidemics in all generations (Table S4). After 6 generations of selection only on susceptibility and genetic variance of 0.2, less than 10% of epidemics occurring were long epidemics, whilst only 3 generations of combined selection were required for the same outcome. After 9 generations of combined selection there were no long epidemics occurring, whilst with selection only on susceptibility required 18 generations (Table S4).

**Table S4. Short and long epidemics over generations of selection**

|  | Selection on susceptibility | | | | | | Selection on susceptibility and infectivity | | | | | |
| --- | --- | --- | --- | --- | --- | --- | --- | --- | --- | --- | --- | --- |
| Generation | **No epidemic** | **se** | **Short** | **se** | **Long** | **se** | **No epidemic** | **se** | **Short** | **se** | **Long** | **se** |
| 0 | 0.38 | 0.01 | 0.21 | <10^-2^ | 0.21 | 0.01 | 0.38 | 0.01 | 0.21 | <10^-2^ | 0.21 | 0.01 |
| 1 | 0.40 | 0.01 | 0.23 | 0.01 | 0.19 | 0.01 | 0.42 | 0.01 | 0.26 | 0.01 | 0.17 | <10^-2^ |
| 2 | 0.41 | 0.01 | 0.27 | <10^-2^ | 0.17 | <10^-2^ | 0.46 | 0.01 | 0.31 | 0.01 | 0.11 | <10^-2^ |
| 3 | 0.45 | 0.01 | 0.27 | 0.01 | 0.14 | <10^-2^ | 0.52 | 0.01 | 0.32 | 0.01 | 0.07 | <10^-2^ |
| 4 | 0.48 | 0.01 | 0.29 | 0.01 | 0.12 | <10^-2^ | 0.57 | 0.01 | 0.32 | 0.01 | 0.04 | <10^-2^ |
| 5 | 0.48 | 0.01 | 0.31 | 0.01 | 0.10 | <10^-2^ | 0.61 | 0.01 | 0.32 | 0.01 | 0.02 | <10^-2^ |
| 6 | 0.50 | 0.01 | 0.32 | 0.01 | 0.08 | <10^-2^ | 0.64 | 0.01 | 0.31 | 0.01 | 0.01 | <10^-2^ |
| 7 | 0.53 | 0.01 | 0.32 | 0.01 | 0.06 | <10^-2^ | 0.68 | 0.01 | 0.29 | 0.01 | 0.01 | <10^-2^ |
| 8 | 0.56 | 0.01 | 0.33 | 0.01 | 0.05 | <10^-2^ | 0.72 | 0.01 | 0.25 | 0.01 | 0.01 | <10^-2^ |
| 9 | 0.58 | 0.01 | 0.32 | 0.01 | 0.04 | <10^-2^ | 0.76 | 0.01 | 0.22 | 0.01 | 0.00 | <10^-2^ |
| 10 | 0.60 | 0.01 | 0.32 | 0.01 | 0.03 | <10^-2^ | 0.78 | <10^-2^ | 0.20 | <10^-2^ | 0.00 | <10^-2^ |
| 11 | 0.60 | 0.01 | 0.33 | 0.01 | 0.03 | <10^-2^ | 0.83 | 0.01 | 0.16 | 0.01 | 0.00 | <10^-2^ |
| 12 | 0.64 | 0.01 | 0.30 | 0.01 | 0.02 | <10^-2^ | 0.85 | 0.01 | 0.14 | 0.01 | 0.00 | <10^-2^ |
| 13 | 0.65 | 0.01 | 0.30 | 0.01 | 0.01 | <10^-2^ | 0.86 | 0.01 | 0.13 | 0.01 | 0.00 | <10^-2^ |
| 14 | 0.67 | 0.01 | 0.28 | 0.01 | 0.01 | <10^-2^ | 0.89 | <10^-2^ | 0.10 | <10^-2^ | 0.00 | <10^-2^ |
| 15 | 0.69 | 0.01 | 0.27 | 0.01 | 0.01 | <10^-2^ | 0.91 | <10^-2^ | 0.08 | <10^-2^ | 0.00 | <10^-2^ |
| 16 | 0.72 | 0.01 | 0.25 | 0.01 | 0.00 | <10^-2^ | 0.93 | <10^-2^ | 0.07 | <10^-2^ | 0.00 | <10^-2^ |
| 17 | 0.75 | 0.01 | 0.23 | 0.01 | 0.01 | <10^-2^ | 0.93 | <10^-2^ | 0.06 | <10^-2^ | 0.00 | <10^-2^ |
| 18 | 0.75 | 0.01 | 0.23 | 0.01 | 0.00 | <10^-2^ | 0.94 | <10^-2^ | 0.05 | <10^-2^ | 0.00 | <10^-2^ |
| 19 | 0.76 | 0.01 | 0.22 | 0.01 | 0.00 | <10^-2^ | 0.95 | <10^-2^ | 0.04 | <10^-2^ | 0.00 | <10^-2^ |
| 20 | 0.78 | 0.01 | 0.20 | 0.01 | 0.00 | <10^-2^ | 0.96 | <10^-2^ | 0.04 | <10^-2^ | 0.00 | <10^-2^ |

*In the table, selection is either only on susceptibility with accuracy 0.7, or on both susceptibility and infectivity with accuracies 0.7.*
