# Supplementary material for: Enhancing genetic disease control by selecting for lower host infectivity and susceptibility
Source: Heredity (Edinb). 2019 Jan 16;122(6):742–58. doi: 10.1038/s41437-018-0176-9 (PMC6781107; doi:10.1038/s41437-018-0176-9)
Supplement: Supplementary file 5 — Supplementary Information 5 [file 41437_2018_176_MOESM5_ESM.docx]

**Supplementary Information 5**

***Impact of genetic correlations between susceptibility and infectivity on response to selection***

*Methods*

To assess the impact of genetic correlations on the epidemiological consequences of selection for host susceptibility and infectivity, populations with correlated true breeding values (TBVs) for susceptibility and infectivity were generated. Those were calculated as functions of sire and dam TBVs and Mendelian sampling terms (see Materials and Methods), all sampled from multivariate normal distributions with mean zero and (co)variances specified by the genetic (co)variance matrix **G** (half of the trait genetic (co)variance for the Mendelian sampling term). Specifically, genetic correlations of -0.5, 0 and 0.5 were simulated between susceptibility and infectivity.

A selection index was then used to explore the impact of genetic correlations between susceptibility and infectivity on response to selection and epidemic characteristics. Response to selection in each trait was calculated as$R=\frac{i \times\mathbf{b}^{T}\times\mathbf{G}_{\boldsymbol{j}}}{\sqrt{\mathbf{b}^{T} \times\mathbf{P}\times\mathbf{b}}}$, where i is the selection intensity for the index (here assumed 50% selection, i.e. i=0.798), **b** is the vector of weights applied to each of the traits in the index (here assumed to be either 0.5 for both traits, or 1 for susceptibility and 0 for infectivity), **G*_j_*** is the j^th^ column of the genetic (co)variance matrix **G** for trait *j*, and **P** is the phenotypic (co)variance matrix ([Cameron, 1997](#_ENREF_7)). The phenotypic variances and covariances for each trait were calculated as the sum of the genetic and environmental variances and covariances, respectively.

*Results*

Compared to the case of independent traits, a favourable genetic correlation between susceptibility and infectivity accelerated selection response in epidemic risk and severity when selection was on susceptibility alone. For example, assuming a favourable genetic correlation of 0.5 between susceptibility and infectivity and zero environmental correlation, genetic variances of 0.5 for both traits and selection intensity of 0.5 for the index, selection only on susceptibility required 6 generations to reduce the risk of epidemics by 50% (Fig. S5), and 3 generations to reduce the proportion of infected individuals by 50%. Notably, selection on susceptibility alone, assuming that susceptibility and infectivity are independent traits, required 13 generations for reducing the proportion of occurring epidemics by at least 50% (see Results, Fig. 4), and 6 generations for reducing the average proportion of infected individuals by at least 50% (Results, Fig. 5). Including infectivity in the selection index was found to be less important when susceptibility and infectivity were favourably correlated; selection considering both susceptibility and infectivity with equal weights of 0.5 performed only marginally better than selection only on susceptibility, requiring 5 generations to reduce the proportion of epidemics that occurred by 50% (Fig. S5), and 3 generations to reduce the proportion of infected individuals by 50%.

With a negative genetic correlation of -0.5 between susceptibility and infectivity progress in reducing epidemic risk and severity was substantially delayed: selection only on susceptibility required 18 generations to reduce the proportion of epidemics by 50%. However, selection combining susceptibility and infectivity helped overcome this delay and required 12 generations (Fig. S5). In other words, with a negative genetic correlation, including both susceptibility and infectivity reduced the delay due to indirect correlated responses in infectivity by 6 generations.

These results suggest that a breeding scheme directly selecting only for susceptibility and ignoring infectivity, could suffer a substantial loss in response to selection due to unfavourable correlated responses in infectivity. The estimates of number of generations that are presented here, are likely to be over-predictions, as the approach followed is a simplification assuming high selection accuracy and not accounting for the loss of variance due to selection. Nevertheless, the comparison between selection only for reduced susceptibility and combined selection for both susceptibility and infectivity, provides a general idea of the relative gains expected by considering both traits in breeding schemes given their genetic correlations.

**Figure S5. Change in epidemic risk over generations of selection assuming that susceptibility and infectivity are genetically correlated**

*Impact on epidemic risk of positive genetic correlation cor_G_=0.5 (left), and negative genetic correlation cor_G_=-0.5 (right) between susceptibility and infectivity, with index selection (a) only on susceptibility by assuming in the index a weight of zero for infectivity (black line), and (b) on both susceptibility and infectivity with equal weights.*

*
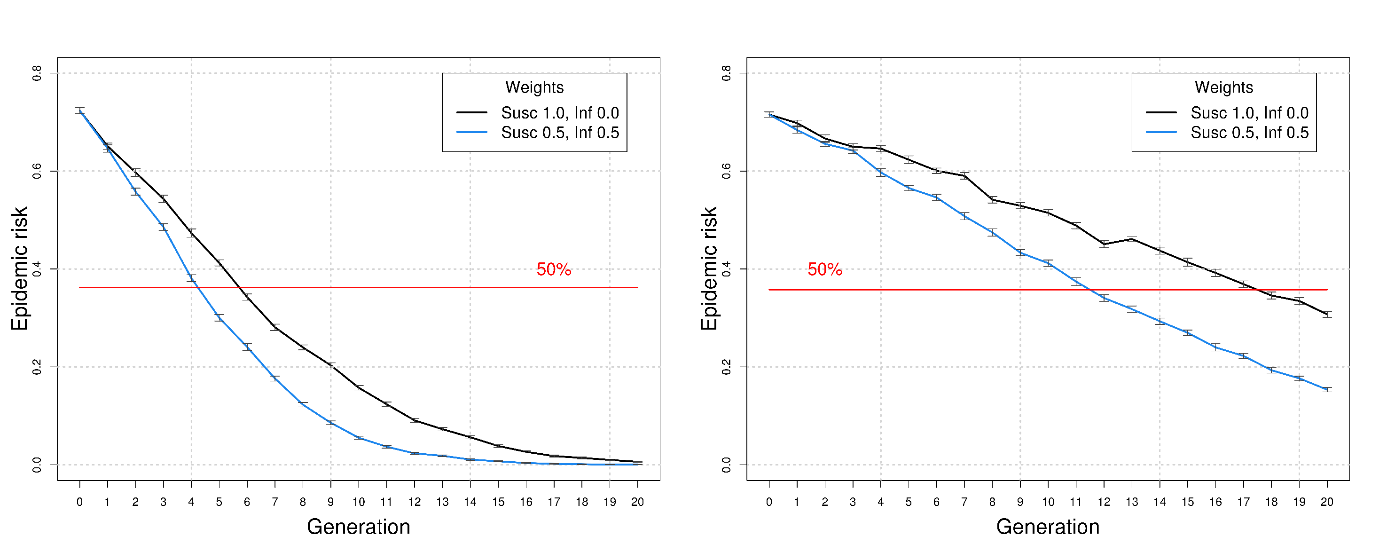
*
