# Supplementary material for: Enhancing genetic disease control by selecting for lower host infectivity and susceptibility
Source: Heredity (Edinb). 2019 Jan 16;122(6):742–58. doi: 10.1038/s41437-018-0176-9 (PMC6781107; doi:10.1038/s41437-018-0176-9)
Supplement: Supplementary file 6 — Supplementary Information 6 [file 41437_2018_176_MOESM6_ESM.docx]

**Supplementary Information 6**

***Assessing the impact of group size on epidemic risk after selection***

Alternative scenarios were tested where the size of the contemporary group was 20 individuals, assuming 50% selection on sires for both susceptibility and infectivity. As shown in Figure S6, with smaller group size, the differences between scenarios in terms of number of generations required to bring R_0_ below 1 were less pronounced. However, there was a significant difference between scenarios for the number of generations required to achieve disease eradication.

More specifically, for the genetic variance of 0.5, selection only on susceptibility required 5 generations for reducing R_0_ from 3.28 to <1, whilst 3 generations of combined selection were sufficient to bring R_0_ below 1. For the genetic variance of 0.2 and selection only on susceptibility, R_0_ was reduced from 1.37 to <1 in 3 generations, whilst combined selection required 2 generations to achieve the same outcome.

**Figure S6. Change in the population R_0_ over generations of selection for the group size of 20 individuals**

*The graphs show the change per generation in the population R_0_ for different genetic variances. The black line represents selection only on susceptibility (r_susc_=0.7); the blue line represents combined selection on susceptibility and on infectivity (r_susc_=r_inf_=0.7). The vertical bars represent standard errors over 50 replicates. The red line shows the R_0_ threshold value of 1.*

*
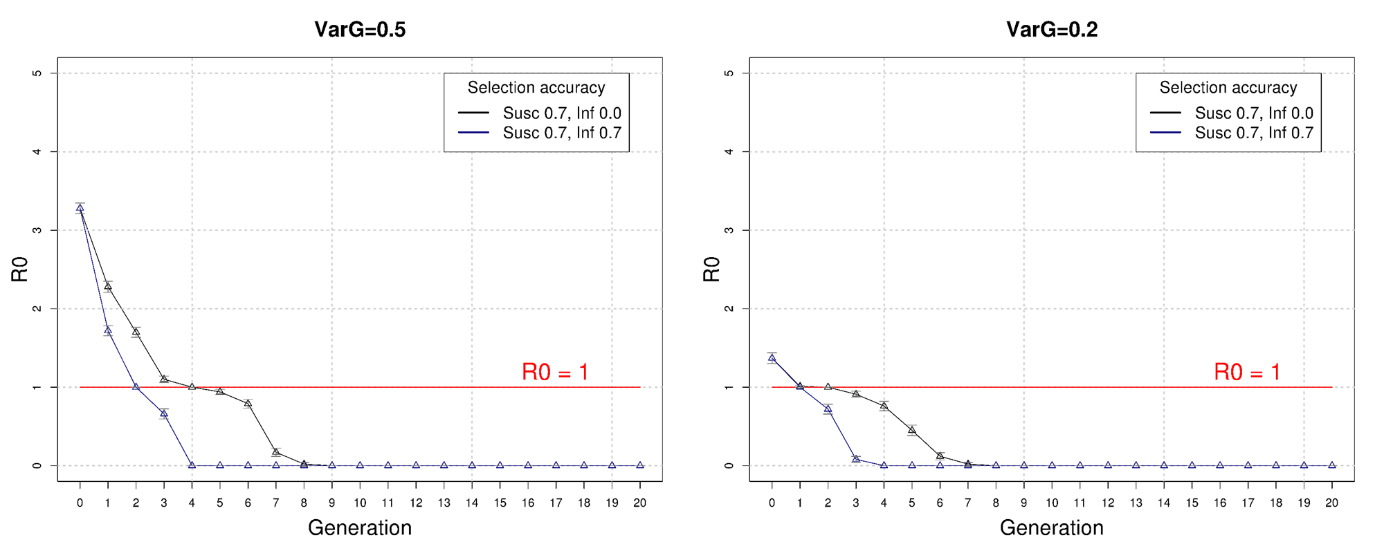
*
